# Analytical and Clinical Performance of High-Sensitivity Cardiac Troponin Point-of-Care Assays as an Aid in the Diagnosis of Myocardial Infarction: A Narrative Review

**DOI:** 10.1155/emmi/5717892

**Published:** 2025-11-14

**Authors:** Lucie Blanc, Ambrine Vaissaire, Nathalie Renard, Cathinca Vargmo, Gro Leite Størvold, Ania Bouhadef, Pierre-Géraud Claret

**Affiliations:** ^1^Global Medical Affairs Department, bioMérieux S.A., Marcy-l'Étoile, France; ^2^Global Marketing Department, bioMérieux S.A., Marcy-l'Étoile, France; ^3^Immunoassay Research and Development Department, bioMérieux S.A., Marcy-l'Étoile, France; ^4^Global Clinical & Regulatory Affairs Department, bioMérieux S.A., Marcy-l'Étoile, France; ^5^Hospices Civils de Lyon, Édouard Herriot University Hospital, Lyon, France

**Keywords:** accelerated diagnostic protocol, acute coronary syndrome, cardiac troponin, chest pain, high-sensitivity, myocardial infarction, point-of-care systems

## Abstract

**Background:**

Acute coronary syndrome (ACS) poses a significant burden worldwide; however, the development of high-sensitivity cardiac troponin (hs-cTn) assays has greatly improved patient management by enabling the detection of very low levels of troponin. The objective of this review was to identify current hs-cTn point-of-care (POC) assays, describe their key features, and discuss their analytical and clinical performance.

**Methods:**

PubMed, MEDLINE, and Embase databases, as well as relevant web sources, were searched for publications up to April 10, 2025. The references included describe the main characteristics of POC hs-cTn assays and their companion instruments, as well as studies assessing their analytical or clinical performance in the context of acute myocardial infarction diagnosis.

**Results:**

In addition to information publicly available from the web, 27 publications were considered relevant for this review. From the retrieved sources, seven POC hs-cTn assays were identified as currently cleared by the United States Food and Drug Administration or CE-marked. Four additional POC hs-cTn assays, each evaluated for analytical or clinical performance, were identified as currently or previously under development. POC instruments differ in their key characteristics, many of which are crucial for ensuring their suitability in specific clinical settings and intended applications. Despite some variability in performance across different platforms, they are generally consistent with the high-sensitivity profile expected of cTn assays. Clinical performance indicators for hs-cTn assays align with European Society of Cardiology (ESC) recommendations, particularly when ESC-recommended diagnostic algorithms are applied. Reported sensitivity and negative predictive values exceed 99%, while positive predictive values are above 70%. Moreover, comparative studies of POC hs-cTn assays and laboratory-based hs-cTn tests have demonstrated no significant differences in diagnostic accuracy for ruling in or ruling out acute myocardial infarction.

**Conclusion:**

POC hs-cTn assays represent a promising alternative to traditional laboratory testing, providing similar analytical and clinical performance while enabling faster diagnosis and management of ACS. Expanded use of hs-cTn assays in clinical practice could transform patient care pathways, especially in time-critical situations. Continued research and ongoing technological advancements are critical to ensure optimal use and widespread adoption in routine clinical settings.

## 1. Introduction

Chest pain is a prevalent symptom that leads to emergency department (ED) visits worldwide. Nontraumatic chest pain represents approximately 5% of all adult visits [1, 2], accounting for over 8 million ED visits annually in the United States of America (USA). Studies indicate that more than 55% of patients presenting with chest pain in the ED are ultimately diagnosed with noncardiac chest pain [1, 3, 4]. Nevertheless, 2%–4% of chest pain cases are attributed to acute coronary syndrome (ACS), a clinical diagnosis that includes ST-segment elevation myocardial infarction (MI) (STEMI) and non-ST–elevation ACS (NSTE-ACS). STEMI is confirmed by electrocardiogram (ECG) findings and transient elevation of cardiac troponin (cTn) (including cTnI or cTnT isoforms) levels [5]. Patients with a working diagnosis of NSTE-ACS are further classified as having non-ST–STEMI (NSTEMI) or unstable angina (UA), which can be differentiated by an elevation of cTn in the former but not the latter [6]. According to the Fourth Universal Definition of MI, a rise and/or fall of cTn values, with at least one value above the 99^th^ percentile upper reference limits (URLs), indicates acute MI (AMI) when accompanied by clinical evidence of acute myocardial ischemia [7]. Since cTn levels play a key role in the diagnosis of AMI, cTn assays have become the cornerstone of its diagnostic workup [1] over the past decades. They surpass earlier cardiac biomarkers such as myoglobin or creatine kinase–myoglobin binding (CK-MB), which are less specific for myocardial injury. Since the initial development of cTnI and cTnT assays, their sensitivity and analytical performance have significantly improved, leading to highly sensitive (hs) cTn (hs-cTn) assays. The 5th generation hs-cTn T and I assays can detect cTn at concentrations 10- to 100-fold lower than conventional assays [8]. Data from large multicenter studies consistently show that hs-cTn assays improve diagnostic accuracy for AMI at the time of presentation compared with conventional assays [5]. Therefore, these assays are recommended by the international guidelines for the diagnosis and care of patients suspected of having NSTE-ACS over less-sensitive assays [5, 9, 10]. Currently, commercial hs-cTn assays include Access hs-TnI (Beckman Coulter, Inc., Brea, CA, USA), Architect and Alinity hs-TnI (Abbott Laboratories, Abbott Park, IL, USA), Atellica IM TnIH (Siemens Healthcare Diagnostics Inc., Tarrytown, NY, USA), VIDAS hsTroponin I (bioMérieux, Marcy-l'Étoile, France), and Elecsys hs-cTnT (Roche Diagnostics International Ltd., Basel, Switzerland). They are widely used in hospital laboratories but require regular calibration and quality control to maintain performance levels specified in the manufacturers' instructions for use, as well as preanalytical steps. These requirements typically restrict their operation to well-trained laboratory personnel. Moreover, their large size requires substantial space, further limiting utilization outside centralized laboratory settings. Finally, the turnaround time from blood draw to result availability in the laboratory information system using central laboratory analyzers is approximately 1–2 h [11]. This makes it difficult to achieve cTn results consistent with the cadence outlined by the European Society of Cardiology (ESC), in settings such as the ED, which recommend a hs-cTn test result at presentation and a second hs-cTn result at 1 or 2 h following the first.

Considering this, and because AMI represents a rapid, life-threatening manifestation of coronary artery disease [12], point-of-care (POC) cTn assays appear to be a major step forward in the management of patients presenting with chest pain. POC involves diagnostic testing conducted near or at the patient's location, with results provided in a timely manner to the clinician responsible for immediate patient care [13]. POC devices should be user-friendly and capable of using whole blood samples, avoiding the need for sample preparation such as centrifugation and pipetting steps [14], which allows operation by nonlaboratory staff (e.g., nurses, clinicians, or other healthcare professionals [HCPs]). POC devices can be categorized into three types: (1) conventional instruments located in a laboratory space close to acute care areas, which are typically operated by laboratory professionals, and as such, may or may not be classified as POC devices. They adhere to the same standards of training, accreditation, and quality assurance as conventional laboratory equipment; (2) benchtop (or desktop) instruments suited for central clinical laboratories or smaller decentralized workspaces. They are not easily portable and lack autonomous functionality, as they require an external power supply to operate, but are suitable for use outside the hospital setting; and (3) portable instruments, designed for easy transportation by an individual, either by hand or on a cart/trolley. These portable devices can be used at the patient's bedside, placed on a desktop, or deployed in testing scenarios outside a hospital setting [13]. Considering this classification and because hs-cTn tests are recommended by international guidelines over less-sensitive tests, the present review will specifically focus on benchtop and portable POC devices, with a particular emphasis on hs-cTn assays and their analytical and clinical performance.

## 2. Methods

A literature search was conducted using PubMed, MEDLINE, and Embase (latter two through the Ovid portal) databases to identify publications on POCT hs-cTn and (1) identify currently available and emerging POC hs-cTn assays and their corresponding POC instruments, (2) summarize the main characteristics of the identified POC instruments, and (3) review the analytical and clinical performance of the identified POC hs-cTn assays.

Search terms included “troponin”(MeSH) and “point of care system”(MeSH) in PubMed and “point-of-care,” “troponin,” and “high-sensitiv^∗^” in Ovid databases, without restrictions on publication year. The analysis focused on information related to hs-cTn measurement using POC tests and included all sources published before April 10, 2025. All sources were considered, including peer-reviewed articles, systematic reviews, and conference abstracts. In parallel, information available in the 510(k) Premarket Notification database from the US Food and Drug Administration (FDA) and publicly available Web sources (e.g., each company's website, International Federation of Clinical Chemistry Committee on Clinical Application of Cardiac Biomarkers [IFCC C-CB] tables) were reviewed. Only English language sources were included in this study.

The inclusion criteria focused on studies evaluating hs quantitative assays for cTn. Analytical performance studies were selected if they assessed at least one criterion such as the limit of blank (LoB), limit of detection (LoD), limit of quantification (LoQ), 99^th^ percentile URL, or percentage of healthy individuals with a cTn concentration higher than the LoD. Clinical performance studies were included if they evaluated diagnostic criteria for AMI such as sensitivity, specificity, positive predictive value (PPV), and negative predictive value (NPV), at various timepoints after patient presentation or using the hs-cTn–based pathways (i.e., those outlined by the ESC-derived 0/1 h or 0/2 h algorithms). When both derivation and validation cohorts were reported in the same study, only data from the validation cohort were included. The same approach was applied to sample types: when multiple sample types were available (e.g., whole blood and plasma), only results from whole blood were reported; if whole blood data were unavailable, results from other blood-derived specimens (such as plasma or serum) were used. Studies were excluded if they used lateral flow assays due to low sensitivity and limited quantitative capacity [15], if they focused solely on concordance analysis between POC and laboratory-based assays or on nonblood-derived samples (e.g., saliva), or if they included fewer than 100 patients. In addition, qualitative tests assessing cTn with other cardiac markers and pathways incorporating medical history or physical findings (e.g., ADAPT, HEART, or GRACE scores) were excluded. POC cTn tests that did not meet hs criteria were also excluded, such as AQT90 FLEX TnI and TnT (Radiometer Medical ApS, Brønshøj, Denmark), Cobas h 232 (cTnT) (Roche Diagnostics International Ltd., Basel, Switzerland), Stratus CS 200 Acute Care Troponin I (cTnI) (Siemens Healthcare Diagnostics Inc., Tarrytown, NY, USA), and RAMP Troponin I (Response Biomedical Corporation, Vancouver, British Columbia, Canada) [16].

## 3. Results

The search yielded 346 and 560 sources from the PubMed and Ovid databases, respectively. Several sources were indexed in both databases. To manage the large number of Ovid sources, the search was refined to 473 sources by focusing on publications dated 2015 and later.

Based on the screening of available sources, seven POC hs-cTn assays were identified as cleared by the FDA or approved by European Union (EU) authorities (CE-marked). Four additional POC hs-cTn assays were identified as currently or previously in development, based on the studies evaluating their analytical or clinical performance. To the best of our knowledge, these four POC hs-cTn assays have not been cleared or approved by US or EU regulatory authorities, respectively.

### 3.1. POC hs-cTn Assays

#### 3.1.1. Currently Cleared or Approved POC hs-cTn Assays

According to the reference table from the IFCC C-CB [17], six POC cTnI assays reaching the hs criteria defined by the IFCC (ability to measure > 50% of concentrations above the LoD in healthy individuals and an imprecision/coefficient of variation (CV) of less than 10% at sex-specific 99^th^ percentile URL) are considered as POC assays: Atellica VTLi hs-cTnI (Siemens Healthcare Diagnostics Inc., Tarrytown, NY, USA), iStar 500 hs-cTnI (Shenzhen Drawray Biotech Co., Ltd., Shenzhen, China), i-STAT hs-TnI (Abbott POC Inc., Princeton, NJ, USA), PATHFAST hs-cTnI (PHC Corporation, Tokyo, Japan), PATHFAST hs-cTnI-II (PHC Corporation, Tokyo, Japan), and TriageTrue hs Troponin I test (Quidel Corporation, San Diego, CA, USA). Among them, Atellica VTLi hs-cTnI, iStar 500 hs-cTnI, PATHFAST hs-cTnI, and TriageTrue hs-cTnI are CE-marked [16, 18–21], and PATHFAST hs-cTnI-II and i-STAT hs-TnI tests are FDA-cleared (respectively, in March 2024 and January 2025) [22, 23]. Based on the literature analysis, a seventh assay was identified: the hs-cTnI Assay Kit performed on the instrument Surelite 8 (Sansure Biotech Inc., Changsha, Hunan Province, China) instrument, which was CE-marked in November 2023 [24]. The key features of all tests/instruments are listed in Table 1.

Atellica VTLi hs-cTnI is used with the Atellica VTLi analyzer (Siemens Healthcare Diagnostics Inc., Tarrytown, NY, USA), which is the smallest of the six approved instruments. It is a handheld device that includes a battery (allowing up to 60 tests per full charge) and a desktop docking station that can be connected to AC power. This device can analyze only one sample at a time, and currently, one parameter (hs-cTnI) is being validated [25].

The second, iStar 500 hs-cTnI, is used with the mono-test iStar 500 analyzer (Shenzhen Drawray Biotech Co., Ltd., Shenzhen, China). Although the platform is a benchtop analyzer weighing 55 kg, it is classified as a POC system in the IFCC C-CB table [17].

The third, i-STAT hs-TnI test, is used with the i-STAT 1 analyzer (Abbott POC Inc., Princeton, NJ, USA). Its weight and size are comparable to those of the Atellica VTLi analyzer, and it can be handled near the patient's bedside. Only one cartridge can be run at a time in this instrument [22, 26].

PATHFAST hs-cTnI and PATHFAST hs-cTnI-II tests are used with PATHFAST (PHC Corporation, Tokyo, Japan), one of the biggest instruments identified in this review (weight: 28 kg) [23, 27]. Both PATHFAST hs-cTnI and PATHFAST hs-cTnI-II are considered hs [28]; while PATHFAST hs-cTnI is CE-marked and intended for the EU market, PATHFAST hs-cTnI-II is FDA-cleared and intended for the US market. The two assays use a different standardization, leading to slight differences in analytical performance (Tables 2 and 3) [29].

The sixth test is the TriageTrue hs Troponin I test (TriageTrue hs-cTnI), which is used with the Triage MeterPro device (Quidel Corporation, San Diego, USA). It is portable because of its low weight and ability to run approximately 100 tests on battery power. It can be used to analyze only one sample at a time [30].

The hs-cTnI Assay Kit working with Surelite 8 (Sansure Biotech Inc., Hunan Province, China), which is not reported in the IFCC C-CB table, is the fifth CE-marked assay and is performed on a desktop analyzer [31].

The intended use for all tests includes the diagnosis of MI or ACS, except for the iStar 500 hs-cTnI and Surelite 8 hs-cTnI assays; no intended use information was available for these assays. Risk stratification of patients presenting with suspected ACS was also included in the intended use for PATHFAST hs-cTnI and PATHFAST hs-cTnI-II.

#### 3.1.2. Emerging POC hs-cTn Assays

Based on published scientific literature, four additional tests, capable of measuring cTn with hs, have been identified as currently or previously in development; however, they have not been cleared or approved by US or European regulatory authorities, respectively. Some of these tests used CE-marked instruments [32, 33].

The SpinChip analyzer (bioMérieux, Marcy-l'Étoile, France) is a multianalyte platform featuring a unique microfluidic system based on dual-axle centrifugation, a vision system for real-time monitoring, advanced reagent formulations, and two integrated readout systems [34].

The Konica Minolta (KM) SPFS–based POC system (KM Inc., Tokyo, Japan) for hs-cTnI testing consists of a desktop analyzer and disposable test cartridges, which include a sensor chip with immobilized capture antibodies and a reagent chip containing the necessary reagents. This integrated design streamlines the testing process and minimizes manual interventions [35].

The SuperFlex system (Perkin Elmer Diagnostics, Waltham, MA, USA), a CE-marked system, includes a fully automated chemiluminescence analyzer that automates all assay steps, minimizing manual intervention and reducing the potential for human error [32].

The Philips Minicare I-20 system, along with its cTnI blood test (Philips Handheld Diagnostics, Amsterdam, Netherlands), received CE marking in May 2016. It uses a single-use disposable cartridge into which a droplet of blood is directly applied. The system utilizes Philips' proprietary biosensor technology to process the sample [36]. After Philips ended the Minicare project in 2018, the original team created Minicare BV and continued to develop the technology until it was acquired by Siemens in 2019 to finally become the Atellica VTLi hs-cTnI test [37]. Before 2019, several papers evaluating the analytical and clinical performance of this test had been published [38–41]. However, as the Minicare project acquired by Siemens has evolved into the Atellica VTLi, which is discussed in this review, publications on Minicare were not considered in the following tables.

Although reported as a POC instrument in the scientific literature [42, 43], the Pylon hs-cTnI Immunoassay (ET Healthcare, Inc, Palo Alto, CA, USA) is not described in this review because it is not classified as a POC assay, as per the IFCC C-CB tables v062024 [16, 28].

### 3.2. Key Features of Instruments

The key features of instruments are presented in Table 1.

### 3.3. Analytical and Clinical Performance

A total of 27 publications meeting the inclusion criteria and describing analytical and/or clinical performance studies on POC hs-cTn assays were retrieved from PubMed and Ovid databases (see Supporting Table S1).

#### 3.3.1. Analytical Performance

Tables 2 and 3 summarize, for each test, the main key parameters to be considered for the assessment of their analytical performance [52, 53]: the LoB is the highest measurement result that is likely to be observed (with a stated probability [*α*]) for a blank sample; the LoD is the lowest concentration of cTn in a sample that can be distinguished from the analyte-free sample with a probability of 95% (observed result greater than the LoB with a 95% probability); the LoQ is the lowest amount of cTn that can be quantitatively determined with stated accuracy (e.g., with ≤ 10% or 20% imprecision); the 99^th^ percentile URL is the concentration below which 99% of a healthy reference population falls; the percentage of measurable values in healthy subjects is the percentage of cTn values < 99th percentile URL that can be obtained in a reference population of ostensibly healthy subjects. Other parameters can be considered when assessing the analytical performance of hs-cTn assays (e.g., linearity, reportable range, and analytical specificity). However, they were not reported in the following tables, as they were not consistently described in publications, especially for assays still in development.

Some publications on PATHFAST hs-cTnI are not described in Table 3 because the analytical performance did not meet the main hs criteria as defined by the IFCC [66].

#### 3.3.2. Clinical Performance

**Figure 1:**
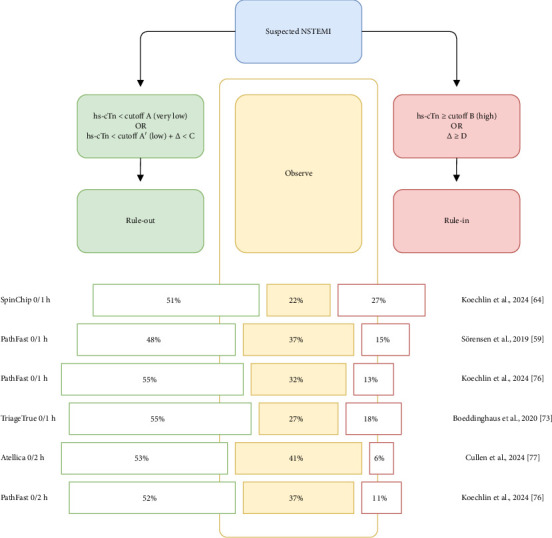
Clinical decision pathway for patients suspected of NSTEMI using ESC-derived 0 h/1 h or 0 h/2 h algorithms with corresponding proportion of patients classified in the categories.

After excluding clinical and ECG signs suggestive of STEMI or very high-risk NSTE-ACS, biomarkers of cardiomyocyte injury, such as cTn, play a complementary role in the diagnosis, risk stratification, and downstream management of patients with suspected ACS [5]. If the clinical presentation aligns with myocardial ischemia, a rise and/or fall in cTn levels exceeding the 99^th^ percentile URL supports a diagnosis of AMI according to the Fourth Universal Definition of MI [7]. In patients with AMI, cTn levels increase rapidly, typically within 1 hour, when using hs assays, following symptom onset, and remain elevated for several days. Based on these kinetics, and because of the higher sensitivity of hs-cTn assays, rapid rule-in and rule-out algorithms (i.e., 0 h/1 h algorithm or 0 h/2 h algorithm) are recommended and endorsed by international guidelines [5, 9, 67], even considered as the best options by the ESC. An optimal threshold for rule-out, always lower than the 99^th^ percentile URL, should be selected to allow sensitivity and NPV of at least 99% or 99.5%, respectively. The optimal threshold for rule-in, usually above the 99^th^ percentile URL, should be selected to allow a PPV above 70%. When the ESC 0 h/1 h or 0 h/2 h algorithms are not available, the assay-specific 99^th^ percentile URL can be used as a threshold for AMI diagnosis, particularly valuable with serial samples over a minimum of 4 h and especially in early presenters (symptoms at presentation ≤ 2–3 h). Tables 4 and 5 provide an overview of the clinical performance of the POC hs-cTn assays over time [68, 69], using serial sampling at baseline (patient presentation, 0 h) and at follow-up timepoints (e.g., 1, 2, 3, 4, 6, or 8 h), considering the 99^th^ percentile URL as the cutoff. Tables 6, 7, 8, and 9 provide an overview of the clinical performance of the POC hs-cTn assay using the ESC-derived 0 h/1 h or 0 h/2 h algorithms.  Figure 1, presenting data from Table 9, illustrates the proportion of patients classified into the different categories: rule-out, observation, and rule-in.

Only limited information was available regarding the clinical performance of Surelite 8 hs-cTnI: Yin et al. [62] published, in 2024, the results of a study, which showed sensitivity and specificity for AMI diagnosis as 100% and 92.3%, respectively, with no further details. Regarding SuperFlex hs-cTnI, based on 200 patients with chest pain, of which 40 were adjudicated AMI (prevalence: 20%), Zhang et al. [50] reported sensitivity, specificity, PPV, and NPV of diagnostic performance for AMI diagnosis of 100%, 81.25%, 57%, and 100%, respectively (cutoff = 99^th^ percentile URL; i.e., 25.6 ng/L).

In most studies, major adverse cardiac events (MACEs), with variable definitions including cardiac death at 30 days and all-cause mortality at 2 or 5 years, were assessed primarily to evaluate the safety of ESC-derived triage algorithms. In addition, MACE rates were analyzed to explore the potential role of POC hs-cTn in risk stratification of patients with suspected AMI. Safety has been assessed in several studies involving Atellica VTLi hs-cTnI [72, 74, 75, 77], PATHFAST hs-cTnI [76], TriageTrue hs-cTnI [73], and SpinChip hs-cTnI [64]. Although the proportion of patients ruled out using a single measurement was lower than with algorithm-based approaches (around 30% vs. more than 50%), the rate of MACE ranged from 0.0% [72] to 0.5% [74]. These figures are comparable to those reported for the 0 h/1 h or 0 h/2 h algorithms for the same assay, ranging from 0.1% [77] to 0.8% [64]. In ruled-out patients, the rate of deaths was 0.2% at 30 days and 2.3% at 2 years [76] for PATHFAST hs-cTnI and 0.0% at 30 days, and 1.6% at 2 years [73] for TriageTrue hs-cTnI. At 5 years, the rate of death in ruled-out patients was 5.1% with SpinChip hs-cTnI [64].

**Figure 2:**
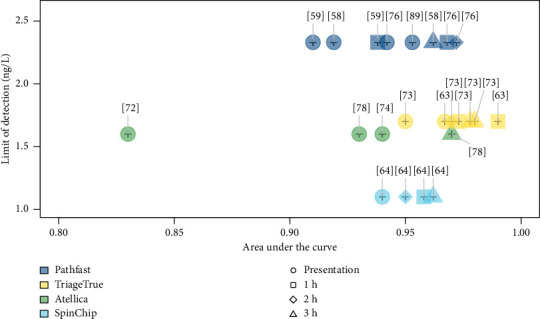
Diagnostic discrimination for hs-cTn and NSTEMI. References of each study evaluating the AUCs of the four tests are presented in brackets .

In several publications, analysis of the area under the receiver operator curve (AUROC), the ability of the test to differentiate between patients with and without AMI was performed for the POC hs-cTn assay and its comparator(s), usually a well-established laboratory-based hs-cTn assay(s).  Figure 2 shows that the area under the curve (AUC) reported for each POC hs-cTn assay at different timepoints correlated with assay-specific LoD.

## 4. Discussion

This review summarizes the analytical and clinical performance of currently available and emerging POC hs-cTn assays. Overall, evidence suggests that these POC assays demonstrate analytical and clinical performance comparable to well-established laboratory-based tests.

Instruments classified as POC devices differ in size and weight, ranging from lightweight handheld systems (approximately 500 g) that can be readily deployed at the patient's bedside to compact small benchtop instruments (5–12 kg) and extending to larger benchtop platforms (up to 28–78 kg), which, although still categorized as POC in a broader sense, are less suited for use in confined clinical environments. This distinction is important when assessing their real-world applicability. Although an intravenous line is typically placed in patients suspected of having NSTE-ACS, the ability to collect blood via capillary sampling offers the advantage of a reduced number of handling steps. Although this is possible with both Atellica VTLi and SpinChip devices, the Atellica VTLi cartridge does not incorporate the system required for direct capillary blood collection from the fingertip. Conversely, SpinChip enables direct sampling into the cartridge, minimizing the risk of cross-contamination with the external environment and accidental blood exposure for HCPs. However, capillary sampling may be affected by suboptimal conditions, particularly in emergency settings (e.g., skin disinfection and cellular debris), which require appropriate training of HCPs. Another key factor is the need for preliminary steps, such as calibration or quality control, which ensures optimal clinical performance, and must be performed according to the manufacturer's requirements. Most POC devices require such manual and periodic procedures before results can be reported, which can be a significant limitation, as these devices are intended for users who may not have a background in clinical laboratory analysis. Importantly, some emerging instruments address this challenge by eliminating the need for these preliminary steps. In many countries, regulations require that POC testing be supervised, and its results properly integrated into laboratory systems, tasks that are typically performed by laboratory staff. The maintenance of these instruments adds to the workload of HCPs, who must organize and update their workflows accordingly. In addition, because POC devices are also designed for use in mobile environments, such as ambulances, where movement and vibrations can occur, it is crucial to consider a certain level of vibration and to ensure correct device functionality under such conditions. For POC devices to be suitable for use in ambulances or other mobile environments, they must be able to operate reliably on internal or external batteries. While some analyzers can perform 60–100 tests per charge, performance and portability remain important considerations when evaluating broader implementation. Another key factor to consider is the instrument's capability to simultaneously analyze multiple parameters (such as D-dimer or NT-proBNP), thereby expediting the patient's final diagnosis and reducing overall costs by using a single cartridge/test, as well as the automatic recognition of sample type. The SpinChip analyzer automatically recognizes the sample type, whereas in the Atellica VTLi analyzer, for instance, the operator must select the sample type, leading to a potential risk of operator errors affecting the results. Finally, the TTR of POC hs-cTn assays ranges from 8 to 20 min, which is substantially shorter than 1–2 h typically observed for laboratory-based assays [11]. This accelerated TTR improves the availability of hs-cTn results in clinical practice, with studies reporting a time saving of up to 70 min compared with central laboratory testing [71, 78].

The analytical characteristics of the different hs-cTnI assays, highlighted by several key parameters such as LoB, LoD, and LoQ at different CVs, 99^th^ percentile URLs, and corresponding CVs or percentages of healthy individuals measured greater than LoD, are presented in this review for both approved and nonapproved assays. The assays vary in terms of LoB and LoD. For instance, KM hs-cTnI, iStar 500 hs-cTnI, SpinChip hs-cTnI, and PATHFAST hs-cTnI exhibit some of the lowest LoD values (approximately 1.0 ng/L), indicating a hs. In contrast, Surelite 8 hs-cTnI has a higher LoD (3.6 ng/L). At a CV of 10%, some assays, such as Surelite 8 hs-cTnI (18 ng/L) and PATHFAST hs-cTnI-II (14.2 ng/L), require higher concentrations to achieve acceptable precision. Others, such as the SpinChip hs-cTnI (3.7 ng/L), display lower LoQ values, indicating better precision at low concentrations. In clinical practice, interpretation of cTn results relies on the assay-specific 99^th^ percentile. Thus, while absolute cutoff values differ due to assay characteristics (e.g., 12 ng/L for the KM hs-cTnI cartridge and 40 ng/L for the Surelite 8 hs-cTnI), the criterion for an elevated result remains consistent. Heterogeneity in reference values may be linked to differences in cohort selection methods and variations in the study populations. According to the hs criteria, an assay should detect cTn above the LoD in at least 50% of healthy individuals. Most assays meet this criterion, but there are variations between sexes. Although these variations have been largely described [79, 80], this can potentially lead to a potential underdetection of AMI in the female population. A limited number of studies complied with the IFCC recommendations to determine the 99^th^ percentile in a cohort consisting of 400 women and 400 men [81]. This noncompliance can result in insufficient statistical power, hindering the calculation of the 95% confidence interval (CI) and, consequently, the generalization of diagnostic thresholds.

Tables 4 and 5 compare the clinical performance of different POC hs-cTnI assays in diagnosing AMI in patients with chest pain at different timepoints and using the 99^th^ percentile as the cutoff. Sensitivity varied across assays, ranging from 64.8% to 100% at baseline (T0 h), with most assays improving over time, reinforcing the importance of serial measurements in AMI diagnosis when combined with suggestive clinical signs and ECG findings. These findings align with previous literature, which supports the role of serial hs-cTnI testing in the identification of myocardial injury using the 99^th^ percentile URL as the cutoff [82, 83]. Despite the high specificity of most assays (> 80%), it declined at later timepoints, raising concerns about differentiating AMI from other conditions. Sex differences were noted in the i-STAT hs-TnI test, with females showing higher sensitivity than males. This discrepancy may be attributed to physiological differences in baseline cTnI levels and the impact of sex-specific thresholds, highlighting the need for sex-specific reference values in hs-cTnI interpretation as recommended by international guidelines [10, 84]. A key finding of this analysis is the consistently high NPV (> 95%) across all assays, suggesting that hs-cTnI testing is particularly effective for ruling out AMI, whereas a low PPV indicates that elevated cTnI levels above the 99^th^ percentile URL alone are insufficient for AMI diagnosis.

The sensitivities, NPVs, and PPVs reported in Tables 4 and 5, do not reach the predefined thresholds recommended by the ESC or by clinical guidelines for the derivation and validation of accelerated algorithms (0 h/1 h or 0 h/2 h algorithms), namely, a minimum of 99% for sensitivities, 99% or 99.5% for NPVs, and 70% for PPVs [5]. These specific clinical decision pathways (CDPs) provided greater precision in the clinical diagnosis of AMI, as shown in Tables 6, 7, and 8. The clinical performance of POC hs-cTnI assays, when used in such CDPs, highlights the variability in sensitivity, specificity, PPV, and NPV, depending on the testing approach. A single measurement at presentation (Tables 6 and 7) generally achieves a hs and NPV for all assays. The study by Apple et al. [74] highlights that using a single measurement at presentation provides a better diagnostic accuracy for ruling out AMI when patients present with chest pain over 2 or 3 h, which is in line with ESC recommendations [5]. In patients presenting earlier, serial measurements of cTn may be clinically relevant [5, 10]. The ESC-derived 0 h/1 h and 0 h/2 h algorithms substantially improve diagnostic accuracy by incorporating serial measurements, enhancing both sensitivity and specificity. Notably, except Atellica VTLi hs-cTnI assay [77], using the accelerated algorithm achieved a sensitivity greater than 99%, and a NPV around 100%. Moreover, the percentage of patients ruled out using a single measurement varied considerably across assays, ranging from 17.8% (Atellica VTLi) to 61.9% (TriageTrue). In contrast, the application of the ESC-derived algorithms increased the proportion of ruled-out patients, around 50%, while maintaining high NPV and a low rate of MACE, thereby improving both safety and efficiency in AMI diagnosis. These findings highlight the limitations of relying solely on a single hs-cTnI measurement at presentation, particularly among early presenters.

The low and high cutoffs were chosen to achieve a sensitivity or an NPV above 99% or 99.5%, along with a PPV exceeding 70%. These performance targets are met in nearly all the studies reviewed (Table 9). As shown in Figure 1, the proportion of patients classified into the observation zone is one of the main criteria distinguishing the different assays: the lower this proportion, the shorter the patient's length of stay in the ED, and the lower the level of clinical uncertainty for the physician. This is particularly the case with the SpinChip assay, which places only 22% of patients under observation [64].

In addition, all assays comply with the acceptable miss rate of 30-day MACE in low-risk patients triaged by 0 h/1 h or 0 h/2 h algorithms, which are generally considered to be less than 1% [85]. These rates are comparable or even superior to those described for well-established laboratory-based tests [86, 87]. This supports the safety and prognostic validity of the algorithms in ruling out serious cardiac events. In conclusion, combining diagnostic accuracy metrics with clinical follow-up data helps to confirm that ESC-derived algorithms with POC assays are both effective and safe, ensuring that low-risk patients can be discharged without compromising care, and supporting their evidence-based use in emergency settings.

Figure 2 demonstrates that all AUROC values, except for Atellica VTLi in one study [72], range between 0.90 and 1.00, indicating a strong ability of the tests to accurately distinguish MI cases from non-MI cases. These values generally rise with the duration from the patient's presentation to the ED, which is consistent with the findings previously discussed. When combined with a very low LoD, these AUROC values emphasize the hs of the currently available or emerging POC hs-cTn assays.

In all the selected publications, the clinical performance of POC hs-cTn assays was compared with that of well-established laboratory-based hs-cTn assays using instruments such as the Architect hs-cTnI (Abbott Diagnostics, Abbott Park, IL, USA), Access hs-cTnI (Beckman Coulter Inc., Brea, CA, USA), Atellica IM hs-cTnI (Siemens Healthcare Diagnostics Inc., Tarrytown, NY, USA), and Elecsys hs-cTnT (Roche Diagnostics International Ltd., Basel, Switzerland). Several comparative methods can be employed to demonstrate the noninferiority of POC assays relative to reference standards. In several studies, DeLong's test [88], a widely adopted nonparametric approach for comparing AUROC values, was used, and no significant difference was observed between the AUROCs of POC hs-cTn assays and reference standards [39, 64, 73, 78, 89, 90]. Moreover, the diagnostic accuracy of the TriageTrue hs-cTnI was superior to that of the two well-established laboratory-based tests [63].

This review has several limitations. First, despite a rigorous search methodology, some eligible studies may have been missed. In particular, no study meeting our search criteria was identified for the i-STAT hs-TnI test using the i-STAT 1 system. Second, we were not always able to confirm whether the identified nonapproved POC hs-cTn assays were still under development at the time of analysis, especially for POC assays described in studies published more than four or 5 years ago with no subsequent publications. Third, some key data, particularly those needed to classify the tests as hs, were sometimes missing or incomplete. For instance, the requirement to detect cTn above the LoD in more than 50% of healthy individuals, in both women and men separately, is often unspecified. Fourth, the review did not address the assessment of the clinical performance of POC hs-cTn assays used in CDPs incorporating medical history and physical findings (e.g., HEART and TIMI). Fifth, all the data described in this article include both whole blood and plasma, as several studies did not assess whole blood samples. Although correlation coefficients between whole blood and plasma have sometimes been reported to be close to 1 [64], the results cannot always be directly compared. Finally, for certain studies, only the abstract was available without access to the full publication, which may have limited the depth of our assessment (see Supporting Table S1).

As shown in this review, the implementation of POC hs-cTn testing offers significant potential to streamline patient management by reducing the time to diagnosis and enabling earlier risk stratification while maintaining analytical and clinical performance comparable to that of laboratory-based assays with confirmed safety outcomes. This may facilitate earlier discharge decisions, alleviate ED overcrowding, and enhance hospital resource allocation, ultimately reducing both length of stay and healthcare costs [91–93]. These benefits are particularly relevant in resource-limited settings such as rural hospitals and mobile healthcare units, where access to centralized laboratory testing is limited [94, 95].

However, certain challenges remain, many of which are inherent to cTn testing. These include a lack of harmonization and standardization of cTn thresholds across different assays. Some studies have investigated the use of machine-learning algorithms specifically designed for POC assay [96, 97], thereby eliminating the need for assay-specific thresholds and demonstrating promising clinical performance. However, randomized clinical trials and studies aimed at assessing the routine clinical implementation of these algorithms should be further conducted. In addition, to achieve the best diagnostic performance in the diagnosis of AMI and risk stratification of patients with chest pain, the need for a second cTn measurement at 1 or 2 h persists for patients who do not meet single low-risk or rule-out criteria. This raises logistical considerations, depending on the location of the POC device, particularly if a second measurement is conducted using a laboratory-based instrument.

## 5. Conclusion

The development of POC hs-cTn assay testing is progressing rapidly, and as highlighted in the various studies reviewed in this article, several POC hs-cTns achieve the necessary sensitivity and precision to detect cTn at very low concentrations when using ESC-derived algorithms, meeting the criteria established by clinical guidelines. Although their diagnostic performance is comparable to that of well-established laboratory-based assays, the key features of POC instruments must be carefully considered to ensure their optimal use and broad acceptance in routine clinical practice.

## Figures and Tables

**Table 1:** Overview of key features of the POC hs-cTn instruments.

Reagent/assay	Instrument	Technology	Sample		TTR	Dimensions and weight of the instrument
			Matrix	Volume		
*Assays currently cleared or approved*						
Atellica VTLi hs-cTnI [44]	Atellica VTLi analyzer	Magnotech Technology	Venous and capillary WB/plasma	30–100 µL	∼8 min	Handled analyzer: 25 × 5.2 × 8.5 cm; W: 780 g Base: 29 × 6 × 10 cm W: 460 g
iStar 500 hs-cTnI [21]	iStar 500 analyzer	CLEIA	Venous WB/plasma/serum	5–150 µL	11 min	Desktop analyzer: 55 × 57 × 68 cm W: 55 kg
i-STAT hs-TnI test [45, 46]	i-STAT 1 analyzer	ELISA	Venous WB/plasma	22 µL	∼15 min	Handled analyzer: 23.5 × 7.7 × 7.3 cm W: 650 g
PATHFAST hs-cTnI [27]	PATHFAST	CLEIA and Magtration Technology	Venous WB/plasma/serum	100 µL	< 17 min	Desktop analyzer: 34.3 × 56.9 × 47.5 cm W: 28 kg
PATHFAST hs-cTnI-II [27]	Triage MeterPro	Fluorescence IA	WB/plasma	175 µL	< 20 min	Desktop analyzer: 22.5 × 19 × 7 cm W: 700 g
TriageTrue hs-cTnI test [47, 48]						
Surelite hs-cTnI [31, 49]	Surelite 8	CLEIA	WB/plasma/serum	NA	∼15 min	Desktop analyzer: 22.8 × 38.5 × 25.6 cm W: 12 kg

*Assays not currently cleared or approved*						
SpinChip hs-cTnI [34]	SpinChip analyzer	Fluorescence IA (UCNP)	Venous and capillary WB/plasma	14 µL	∼10 min	Desktop analyzer: 21 × 24 × 22 cm W: 5 kg
KM hs-cTnI cartridges [35]	KM SPFS POC system	SPFS	WB/plasma	200 µL	∼15 min	Desktop analyzer (dimensions and weight NA)
SuperFlex hs-cTnI [32, 50, 51]	SuperFlex	CLEIA	WB/serum/plasma	100 µL	< 15 min	Desktop analyzer: 79.2 × 67 × 61.5 cm W: 78 kg

Abbreviations: CLEIA, chemiluminescence enzyme immunoassay technology; ELISA, enzyme-linked immunosorbent assay; hs-cTnI, high-sensitivity cardiac troponin I; IA, immunoassay; KM, Konica Minolta; min, minutes; NA, not available; POC, point of care; SPFS, surface plasmon field–enhanced fluorescence spectroscopy; TTR, time to result; UCNP, upconverting nanoparticles; W, weight; WB, whole blood.

**Table 2:** Overview of analytical performance of the approved POC hs-cTn assays on whole blood samples or other types of samples, as reported by the manufacturers.

Assays	LoB (ng/L)	LoD (ng/L)	LoQ 20% CV (ng/L)	LoQ 10% CV (ng/L)	99^th^ percentile URL (ng/L) [CV, %]		Percent (%) healthy subjects measured ≥ LoD	Number of healthy subjects		Sources
Atellica VTLi hs-cTnI	0.6^∗^	1.6	3.7^∗^	8.9^∗^	23 [6.1^∗^]		Overall: 84^∗^ F: 80; M: 87	NA		Brochure, 2021 [54]; IFCC table^∗^ [17]
					F: 19	M: 27				

iStar 500 hs-cTnI	0.3	0.6	0.9	1.9	17 [2.90]		Overall: 93 F: 91; M: 96	648		IFCC table [17]
					F: 11	M: 19		F: 343	M: 303	

i-STAT hs-TnI	0.8	1.6	2.9	6.9	21 [5.2%]		Overall: ≥ 50	895		510 k dossier, 2025; package insert, 2025 [22, 46]
					F: 13	M: 28		F: 405	M: 490	

PATHFAST hs-cTnI	1.2	2.3	NA	14.2	29 or 28^1^ [6.1 or 6.6^1^]		Overall: 66 F: 53; M: 79	734		Package insert, 2024^2^; Brochure, 2024^2^ [20, 27]
					F: 20	M: 30				
PATHFAST hs-cTnI-II	1.5	3.0	4.1	19^∗^	29 [6.1]			F: 352	M: 382	510 k dossier, 2024; IFCC table^∗^ [17, 23]
					F: 20	M: 30				

TriageTrue hs-cTnI	0.5–0.8	1.5–1.9	2.8–2.8	5.8–6.2	21 [6.5^∗^]		Overall: 72^∗^	789		Package insert, 2020^1^, IFCC table^∗^ [17, 47]
	0.6	1.7	2.8	6.2	F: 14	M: 26		F: 391	M: 398	Brochure, 2020 [48]

Abbreviations: CV, analytical coefficient of variation; F, female; hs-cTn, high-sensitivity cardiac troponin; IFCC, International Federation of Clinical Chemistry and Laboratory Medicine; LoB, limit of blank; LoD, limit of detection; LoQ, limit of quantification; M, male; NA, not available; URL, upper reference limit.

^1^After exclusion of individuals with abnormal NT-proBNP, HbA1c, and eGFR.

^2^Information available only for plasma samples.

^∗^Information provided in the IFCC C-CB table v082025 [17].

**Table 3:** Overview of analytical performance of the POC hs-cTn assays on whole blood samples or other types of samples, as published in the scientific literature.

Assays	LoB (ng/L)	LoD (ng/L)	LoQ 20% CV (ng/L)	LoQ 10% CV (ng/L)	99^th^ percentile URL (ng/L) [CV, %]		Percent (%) healthy subjects measured ≥ LoD	Number of healthy subjects		Sources
*Assays cleared or approved*										
Atellica VTLi hs-cTnI	NA	1.2	NA	6.7	23 [NA]		Overall: 84 F: 80; M: 87	693		Apple et al., 2021^2^ [55]
					F: 18	M: 27		F: 330	M: 363	
	0.6	1.6	3.7	8.9	23 [NA]		Overall: 84 F: 80; M: 87	693		Christenson et al:, 2022 [56]
					F: 18	M: 27		F: 330	M: 363	
	NA		9.8		23 [NA]		Overall: > 50	2144		Hatherley et al., 2024 [57]

PATHFAST hs-cTnI	NA	1.0	2.0	3.0	16 [5]		Overall: 66	119		Spanuth et al., 2015^1^ [58]
								F: 59	M: 60	
	NA	1.0	NA		16 [NA]		Overall: 76	474		Spanuth et al., 2016^2^ [29]
					F: 12	M: 17		F: 236	M: 238	
	1.5	2.9	NA	11.0	24 [NA]		Overall: 78 F: 68; M: 85	474		Sörensen et al., 2019^2^ [59]
					F: 21	M: 27		F: 236	M: 238	
	NA	2.3	2.3 [CV NA]		29 [NA]		NA	129		Osredkar et al., 2021 [60]

PATHFAST hs-cTnI-II	1.2	2.3	4.0	14.2	29 or 28 [6.1 or 6.6]		Overall: 66 F: 53; M: 79	734		Christenson et al., 2018^1^ [61]
					F: 20	M: 30		F: 352	M: 382	
	NA	3.0	NA		28 [NA]		Overall: 75	474		Spanuth et al., 2016 [29]
					F: 25	M: 31		F: 236	M: 238	

Surelite 8 hs-cTnI	2.1	3.6	NA	18.0	40 [6.8]		NA	358		Yin et al., 2024^1^ [62]

TriageTrue hs-cTnI	NA		1.2	8.6	21 [< 9]		NA	NA		Dakshi et al., 2024 [63]

*Assays not cleared or approved*										
SpinChip hs-cTnI	0.6	1.1	1.1	3.7	NA^3^		Overall: 75	NA^3^		Koechlin et al., 2024^2^ [64]

KM hs-cTnI cartridge	0.4	0.6	1.5	3.9	12 [4.8]		Overall: 66 F: 55; M: 77	600		Braga et al., 2019 [35]
					F: 11	M: 21				
	NA				20–36 [NA]		Overall: 84 F: 74; M: 92	470		Wu et al., 2020^2^ [65]
					F: 20–35	M: 20–40		F: 220	M: 250	

SuperFlex hs-cTnI	1.0	1.8	NA	12.0	26 [7:2]		Overall: 83 F: 76; M: 90	620		Zhang et al:, 2021^4^ [50]
					F: 24 [7:4]	M: 27 [7:2]		F: 312	M: 308	

Abbreviations: CV, analytical coefficient of variation; F, female; hs-cTn, high-sensitivity cardiac troponin; IFCC, International Federation of Clinical Chemistry and Laboratory Medicine; LoB, limit of blank; LoD, limit of detection; LoQ, limit of quantification; M, male; NA, not available; URL, upper reference limit.

^1^Unknown type of sample.

^2^Information available only for plasma samples.

^3^Specific 99th percentile URL study ongoing.

^4^Information available only for serum samples.

**Table 4:** Overview of clinical performance of POC hs-cTnI assays at different timepoints in patients suspected of NSTEMI, using the 99^th^ percentile URL as a cutoff, as reported by the manufacturers.

Assay		Timepoint	Sensitivity	Specificity	PPV	NPV	Number of patients (prevalence of MI)	Sources
Atellica VTLi hs-cTnI		T 0 h	65%	86%	29%	96%	1089 (*8.4*%)	Brochure, 2021 [54]
		T 2 h	81%	85%	33%	98%		

i-STAT hs-TnI	Female patients	T 0–1 h	92%	83%	29%	99%	1870 (*6.9*%)	Package insert, 2025 [46]
		*T* > 1–3 h	97%	82%	28%	100%	1799 (*6.6*%)	
		*T* > 3–6 h	97%	78%	32%	100%	724 (*9.7*%)	
		*T* > 6 h	100%	55%	44%	100%	60 (*26.7*%)	
	Male patients	T 0–1 h	79%	84%	40%	97%	1090 (*11.9*%)	
		*T* > 1–3 h	91%	84%	42%	99%	1025 (*11.5*%)	
		*T* > 3–6 h	94%	83%	51%	99%	439 (*15.7*%)	
		*T* > 6 h	92%	57%	42%	95%	47 (*25.5*%)	

TriageTrue hs-cTnI		T 0 h	89%	84%	55%	97%	422 (*17.8*%)	Package insert, 2020^1^ [47]
		T 2–4 h	92%	80%	54%	98%	364 (*20.3*%)	
		T 4–8 h	92%	78%	57%	97%	298 (*24.5*%)	

*Note:* The italic values are prevalence of myocardial infarction (MI).

Abbreviations: h, hour(s); MI, myocardial infarction; NPV, negative predictive value; PPV, positive predictive value; T, timepoint.

^1^Information available only for the plasma samples.

**Table 5:** Overview of clinical performance of POC hs-cTnI assays at different timepoints in patients suspected of NSTEMI, using the 99^th^ percentile URL as a cutoff, as published in the scientific literature.

Assay	Timepoint	Sensitivity	Specificity	PPV	NPV	Number of patients (prevalence of MI)	Sources
Atellica VTLi hs-cTnI	T 0 h	65%	86%	29%	96%	1089 (*8.4*%)	Gunsolus et al., 2022 [70]
	T 2 h	81%	85%	33%	98%		
	T 0 h	100%	93%	25%	100%	585 (*2.4*%)	Ho et al., 2024 [71]
	T 3 h	100%	90%	14%	100%	408 (*1.7*%)	
	T 0 h	60%	93%	21%	99%	966 (*4*%)	De Iuliis et al., 2024 [72]

TriageTrue hs-cTnI	T 0 h	80%	92%	63%	97%	1261 (*14*%)	Boeddinghaus et al., 2020^1^ [73]
	T 1 h	86%	91%	61%	98%		
	T 2 h	92%	91%	60%	99%		
	T 3 h	93%	89%	55%	99%		

*Note:* The italic values are prevalence of myocardial infarction (MI).

Abbreviations: h, hour(s); MI, myocardial infarction; NPV, negative predictive value; PPV, positive predictive value; T, timepoint.

^1^Information available only for the plasma samples.

**Table 6:** Overview of clinical performance of POC hs-cTn assays using a single sample rule-out at presentation of patients suspected of NSTEMI, as reported by the manufacturers.

Assays	Cutoff A Very low (% of pts ruled-out)	Sensitivity	NPV	Number of patients (prevalence of MI)	Sources
*Single measurement at presentation*					
PATHFAST hs-cTnI	< 3 ng/L^1^ 37.2%	100.0%	100.0%	792 (NA)	PI, 2024^1^ [20]; Brochure 2024^1^ [27]
TriageTrue hs-cTnI	< 3 ng/L 45%	100.0%	100.0%	1261 (*14.1*%)	Brochure, 2020 [48]

*Note:* The italic values are prevalence of myocardial infarction (MI).

Abbreviation: MI, myocardial infarction; NA, not available; NPV, negative predictive value; PI, package insert; pts, patients.

^1^Presenting at ≥ 3 h after symptom onset (with no new ECG ischemia).

**Table 7:** Overview of clinical performance of POC hs-cTn assays using a single sample rule-out at presentation of patients suspected of NSTEMI, as published in the scientific literature.

Assays	Cut-off A Very low (% of pts ruled-out)	Sensitivity	NPV	Number of patients (prevalence of MI)	Sources
*Single measurement at presentation*					
SpinChip hs-cTnI	< 3 ng/L 27%	100.0%	100.0%	1102 (14.8%)	Koechlin et al., 2024^1^ [64]

Atellica VTLi hs-cTnI	< 4 ng/L 17.9%	98.9%	99.5%	All patients: 1086 (8.1%)	Apple et al., 2022^3^ [74]
	< 4 ng/L 28.6%	94.1%	98.3%	Early presenters^2^: 210 (8.1%)	
	< 4 ng/L 30.3%	100.0%	100.0%	966 (4%)	De Iuliis et al., 2024 [72]
	< 7 ng/L^4^ 35.8%	99.2%	99.7%	2090 (4.2%)	Pickering et al., 2024^4^ [75]

PATHFAST hs-cTnI	< 3 ng/L^4^ 38.3%	100.0%	100.0%	394 (18.4%)	Sörensen et al., 2019^1^ [59]

TriageTrue hs-cTnI	< 3 ng/L 45%	100.0%	100.0%	1261 (14.1%)	Boeddinghaus et al., 2020 [73]
	< 4 ng/L 61.9%	100.0%	100.0%	1157 (5.9%)	Dakshi et al., 2024 [63]

Abbreviation: MI, myocardial infarction; NPV, negative predictive value; pts, patients.

^1^Information available only for plasma samples.

^2^Presenting at < 2 h after symptom onset.

^3^Data coming from the derivation cohort, since in the validation cohort, only plasma samples were assessed.

^4^Presenting at ≥ 3 h after symptom onset (with no new ECG ischemia).

**Table 8:** Overview of clinical performance of POC hs-cTn assays using the ESC-derived algorithms 0 h/1 h in patients suspected of NSTEMI, as reported by the manufacturer.

Assays	Cutoff A	Cutoff A′	Δ C	Cutoff B	Δ D	Sensitivity [95% CI]	Specificity [95% CI]	PPV [95% CI]	NPV [95% CI]	Number of patients (prevalence of MI)	Sources
	Very low	Low	Δ 0–1 h/Δ 0–2 h	High	Δ 0–1 h/Δ 0–2 h						
	Percentage of patients ruled-out			Percentage of patients ruled-in							
*ESC-derived algorithm 0 h/1* *h*											
PATHFAST hs-cTnI	NA	≤ 4 ng/L	≤ 3 ng/L	≥ 90 ng/L	≥ 20 ng/L	99.1% [96.9–99.9]	96.2% [94.8–97.3]	80.1% [73.7–85.5]	99.7% [98.8–100.0]	1221 (NA)	PI, 2024 [20] and Brochure 2024 [27]
	NA			NA							

Abbreviations: CI, confidence interval; MI, myocardial infarction; NA, not available; NPV, negative predictive value; PI, package insert; PPV, positive predictive value.

**Table 9:** Overview of clinical performance of POC hs-cTn assays using the ESC-derived algorithms 0 h/1 h or 0 h/2 h in patients suspected of NSTEMI, as published in the scientific literature.

Assays	Cutoff A	Cutoff A′	Δ C	Cutoff B	Δ D	Sensitivity [95% CI]	Specificity [95% CI]	PPV [95% CI]	NPV [95% CI]	Number of patients (prevalence of MI)	Sources
	Very low	Low	Δ 0–1 h/Δ 0–2 h	High	Δ 0–1 h/Δ 0–2 h						
	Percentage of patients ruled-out			Percentage of patients ruled-in							
*ESC-derived algorithm 0 h/1 h*											
SpinChip hs-cTnI	< 7 ng/L^1^	< 7 ng/L	< 4 ng/L	≥ 36 ng/L^2^	≥ 11 ng/L	100% [97.7–100]	90.9% [88.3–92.9]	72.9% [66.4–78.6]	100% [99.0–100]	765 (21.3%)	Koechlin et al., 2024 [64]
	*51%*			*27%*							

PATHFAST hs-cTnI	NA	< 4 ng/L	< 3 ng/L	≥ 90 ng/L	≥ 20 ng/L	99.1% [95.1–100]	97.6% [95.8–98.7]	86.5% [77.6–92.8]	99.7% [98.1–100]	610 (18.4%)	Sörensen et al., 2019^3^ [59]
	*48%*			*14.6%*							
	< 3 ng/L^1^	< 4 ng/L	< 3 ng/L	≥ 90 ng/L	≥ 20 ng/L	100% [98.2–100]	96.4% [95.2–97.3]	74.9% [68.3–80.5]	100% [99.5–100]	1532 (13.4%)	Koechlin et al., 2024^3^ [76]
	*55%*			*13%*							

TriageTrue hs-cTnI	< 4 ng/L^1^	< 5 ng/L	< 3 ng/L	≥ 60 ng/L	≥ 8 ng/L	100% [95.9–100]	95.0% [92.5–96.8]	76.8% [67.2–84.7]	100% [98.8–100]	545 (16.2%)	Boeddinghaus et al., 2020 [73]
	*55%*			*18%*							

ESC-derived algorithm 0 h/2 h											
Atellica VTLi hs-cTnI	< 4 ng/L	< 6 ng/L	< 5 ng/L	≥ 60 ng/L	≥ 15 ng/L	98.9% [92.4–99.8]	98.5% [96.6–99.4]	74.5% [62.7–83.6]	99.9% [99.3–100]	All patients: 1796 (4.9%)	*Cullen* et al., 2024 [77]
	*53.3%*			*6.1%*							
	< 4 ng/L	< 6 ng/L	< 5 ng/L	≥ 60 ng/L	≥ 15 ng/L	94.1% [68.0–99.2]	97.7% [95.3–98.9]	54.9% [31.6–76.3]	99.6% [97.1–99.9]	Early presenters^2^	
	NA			NA							

PATHFAST hs-cTnI	< 3 ng/L^1^	< 4 ng/L	< 4 ng/L	≥ 90 ng/L	≥ 55 ng/L	100% [92.9–100]	97.7% [95.4–98.9]	81.6% [66.6–90.8]	100% [98–100]	359 (13.9%)	Koechlin et al., 2024^3^ [76]
	*52%*			*11%*							

*Note:* The italic values are, respectively, the “percentage of patients ruled-out” (2nd column) and the “percentage of patients ruled-in” (3rd column).

Abbreviations: CI, confidence interval; MI, myocardial infarction; NA, not available; NPV, negative predictive value; PPV, positive predictive value.

^1^Presenting at > 3 h after symptom onset.

^2^Presenting within 2 h after symptom onset.

^3^Information available only for plasma samples.

## Data Availability

Data sharing is not applicable to this article as no datasets were generated or analyzed during the current study.

## Supporting Information

Supporting Table S1: Listing of publications for each assay after application of inclusion and exclusion criteria as reported in the Methods section.
