# Supplementary material for: Analytical and Clinical Performance of High-Sensitivity Cardiac Troponin Point-of-Care Assays as an Aid in the Diagnosis of Myocardial Infarction: A Narrative Review
Source: Emerg Med Int. 2025 Nov 14;2025:5717892. doi: 10.1155/emmi/5717892 (PMC12638167; doi:10.1155/emmi/5717892)
Supplement: Supporting Information — Additional supporting information can be found online in the Supporting Information section. [file 5717892.f1.docx]

**Additional file 1**

**Supplementary table S1 – Listing of publications for each assay, after application of inclusion and exclusion criteria as reported in methods section**

|  | Number of PubMed or Ovid Publications | | | |  |
| --- | --- | --- | --- | --- | --- |
|  | Analytical performance | | Clinical performance | | |
| **SpinChip® hs-cTnI (bioMérieux)** | 1 | Koechlin et al., 2024 | | |  |
| **Atellica® VTLi hs-cTnI (Siemens)** | 3 | Apple et al., 2021 ; Christenson et al., 2022 ; Harteley et al., 2024 (abstract). | 8 | Apple et al., 2022 ; Gonsunlus et al., 2022 ;  Cullen et al., 2024 ; De Iuliis et al., 2024 ; Ho et al., 2024 ; Pickering et al., 2024 (research letter) ; Zalama-Sanchez et al., 2024 ; Hatherley et al., 2024 (abstract). |  |
| **i-STAT® hs-TnI test (Abbott)** | 0 |  | 0 |  |  |
| **PATHFAST™ hs-cTnI / PATHFAST® hs-cTnI-II (PHC)** | 5 | Spanuth et al., 2015 and 2016 (abstracts) ; Christenson et al., 2018 ; Sörensen et al., 2019 ; Osredkar et al., 2021. | 3 | Sörensen et al., 2019 ; Gopi et al., 2021 ; Koechlin et al., 2024. |  |
| **Surelite 8** **hs-cTnI (Sansure)** | 1 | Yin et al., 2024 (abstract) | | |  |
| **TriageTrue™ hs-cTnI (Quidel)** | 1 | Dakshi et al., 2024 | 3 | Tsui et al., 2019 ; Boeddinghaus et al., 2020 ; Dakshi et al., 2024. |  |
| **Minicare™ cTnI (Philips)** | 1 | De Theije et al., 2019 (abstract) | 1 | Hausfater et al., 2016 (abstract). |  |
| **KM hs-cTnI (Konica Minolta)** | 2 | Braga et al., 2019 (abstract) ; Wu et al., 2020 (letter to the editor). | 0 |  |  |
| **SuperFlex™ hs-cTnI (Perkin Elmer)** | 1 | Zhang et al., 2021 | | |  |
